# Chronotype Modulates Language Processing-Related Cerebral Activity during Functional MRI (fMRI)

**DOI:** 10.1371/journal.pone.0137197

**Published:** 2015-09-23

**Authors:** Jessica Rosenberg, Martina Reske, Tracy Warbrick, N. J. Shah

**Affiliations:** 1 Institute of Neuroscience and Medicine (INM– 4), Medical Imaging Physics, Forschungszentrum Jülich GmbH, 52425, Jülich, Germany; 2 JARA, Translational Brain Medicine, RWTH Aachen University, 52074, Aachen, Germany; 3 Department of Neurology, University Clinic Aachen, 52074, Aachen, Germany; University Of São Paulo, BRAZIL

## Abstract

**Objective:**

Based on individual daily physiological cycles, humans can be classified as early (EC), late (LC) and intermediate (IC) *chronotypes*. Recent studies have verified that chronotype-specificity relates to performance on cognitive tasks: participants perform more efficiently when tested in the chronotype-specific optimal time of day than when tested in their non-optimal time. Surprisingly, imaging studies focussing on the underlying neural mechanisms of potential chronotype-specificities are sparse. Moreover, chronotype-specific alterations of language-related semantic processing have been neglected so far.

**Methods:**

16 male, healthy ECs, 16 ICs and 16 LCs participated in a fast event-related functional Magnetic Resonance Imaging (fMRI) paradigm probing semantic priming. Subjects read two subsequently presented words (prime, target) and were requested to determine whether the target word was an existing word or a non-word. Subjects were tested during their individual evening hours when homeostatic sleep pressure and circadian alertness levels are high to ensure equal entrainment.

**Results:**

Chronotype-specificity is associated with task-performance and brain activation. First, ECs exhibited slower reaction times than LCs. Second, ECs showed attenuated BOLD responses in several language-related brain areas, e.g. in the left postcentral gyrus, left and right precentral gyrus and in the right superior frontal gyrus. Additionally, increased BOLD responses were revealed for LCs as compared to ICs in task-related areas, e.g. in the right inferior parietal lobule and in the right postcentral gyrus.

**Conclusions:**

These findings reveal that even basic language processes are associated with chronotype-specific neuronal mechanisms. Consequently, results might change the way we schedule patient evaluations and/or healthy subjects in e.g. experimental research and adding “chronotype” as a statistical covariate.

## Introduction

Chronotype-specificity is characterised by individual preferences in sleep and wakefulness that reflect endogenous, self-sustained genetic dispositions [1]. Chronotypes are grouped as intermediate chronotypes (IC) that have the highest prevalence in the population [2], late chronotypes (LC) that go to bed late at night and have difficulties getting up in the morning, and early chronotypes (EC) that tend to wake up at very early hours and find it difficult to remain awake beyond their usual bedtime. When behavioural tasks are administered at individuals’ optimal time of day according to their chronotype, participants are more efficient, e.g. in terms of reaction time performance, than when tested at their non-optimal time of day. This synchrony between the subject’s chronotype-specific optimal time and the time of testing is often referred to as the “*synchrony effect*” [3–6].

Importantly, the individual behavioural aspects of chronotype-specificity have a physiological basis: chronotypes are classified according to the circadian rhythm (i.e. rhythm of about 24 hours) of their biological clock that originates in the suprachiasmatic area (SCA) of the hypothalamus [7]. The SCA elicits circadian signals that promote wakefulness and potentially regulates cognitive output during a normal waking day [8]. Previous studies have determined a time window of approximately three hours during subjective evenings which is characterised by maximal circadian wake promotion, i.e. the existence of a powerful drive for wakefulness between ten to twelve hours after awakening [9, 10]. During this time window, the so-called “*wake maintenance zone*” [9], humans are assumed to be prevented from falling asleep early in the evening hours–although the homeostatic sleep pressure is at its highest level.

Although behavioural studies and chronophysiological findings shed light on chronotype-specificity, the link between performance and the neural mechanisms underlying the synchrony effect remains unclear. Surprisingly, functional brain imaging studies investigating chronotype-specificities are still sparse. A study by Schmidt et al. [9] on perceptual inhibition and executive control assessed time of day modulations of performance on the Stroop-task [11]. During functional magnetic resonance imaging (fMRI), participants were required to name the colour of a visually displayed word while ignoring the meaning of the coloured word. ECs versus LCs were examined during their subjective evening. EC compared to LCs exhibited decreased BOLD (blood oxygenation level dependent) responses in cingulate, insular, parietal and occipital brain regions (i.e., areas involved in conflict resolution). Moreover, LCs, but not ECs, showed increased brain activation in hypothalamic structures that have been reported to be involved in promoting wakefulness [12]. It was concluded that LCs could profit from their ability to cope more efficiently with the increasing time spent awake during the subjective evening hours of a normal waking day. Results of performance in the psychomotor vigilance task (PVT) provide additional support that maintaining attention in the evening was associated with higher activation in LCs than ECs in the SCA that includes the circadian master clock [9]. Based on the findings that ECs exhibited attenuated BOLD responses in PVT-related areas (i.e. mainly left inferior frontal and left middle frontal) as well as in the hypothalamus during their subjective evening, it was concluded that ECs seem to be more vulnerable to the increasing time spent awake across a normal waking day than LCs (see also Taillard et al. [13]). To summarize, chronotype-specificities during the subjective evening were not just reported for behavioural parameters (i.e. reaction times) but also for BOLD activation of task-related and hypothalamic areas.

In the study of chronotype-specific alterations, a crucial cognitive capacity has been neglected thus far: the processing of semantic information. Semantic, long established knowledge of objects, facts, people and word meanings is essential for verbal communication. Semantic associations can be investigated via semantic priming tasks that probe the association between a prime word and a target word (that is presented directly after the prime). Prime and target can be unrelated (UR, “buckler–violin”), directly related (DR, “car–garage”) or indirectly related (IR, “anvil–nail”). Typically, the participant is required to decide whether the target is a real word or a non-word (NW, e.g. “fubber”) by pressing a button (i.e. *lexical decision, [14]*). Research shows that decisions are faster when prime and target are related (i.e. directly and indirectly, [14]. It is postulated that the mental representations are located more closely to each other in the so called mental lexicon compared to situations where prime and target are not related [15].

Numerous neuroimaging studies have investigated the neurofunctional basis of semantic priming in healthy subjects. There are different approaches using a variety of tasks investigating how semantic priming works whereby the corresponding neural correlates of semantic priming are well understood (see e.g. [14, 16–19]). For example, directly linked words were reported to be associated with left-lateralized activation clusters in fronto-temporo-parietal regions while indirect priming led to additional right-hemispheric fronto-parietal activations during lexical decisions (i.e. compared to non-words). Certain neuroanatomical areas that are activated in semantic priming tasks are also activated in tasks that involve semantic processing in general. Specifically, the left superior frontal gyrus (SFG) has been related to visual word processing and interpreting word meanings [20], encoding verbal components of semantic associations in working memory tasks [21], processing of words belonging to different semantic categories [22] and linguistically based mental operations [23]. Moreover, the right SFG is thought to relate to the recognition of semantic relationships between directly related, indirectly related or unrelated words [24, 25]. The left supramarginal gyrus has also been associated with semantic processing; it is strongly connected via nerve fibres to Broca’s and Wernicke’s areas (i.e. classically defined language areas) and is additionally connected to visual and somatosensory cortices. It plays a crucial role in classifying the word’s semantic category [25, 26]. Lastly, activation during semantic tasks of the left postcentral gyrus (i.e. classical somato-sensory area) is associated with increased reading ability of the subjects [27, 28] and with reading of such words that are semantically related to movement [27].

To conclude, semantic processing is crucial for various cognitive functions and therefore is a basic prerequisite for successful management of daily living. While previous work related other cognitive functions to chronotype specificity, no studies investigated whether individual preferences in wakefulness relate to semantic priming capacities. Hence, the aim of the present study was to identify the behavioural and neurofunctional substrates that characterise ECs, LCs and ICs in semantic processing. Noteworthy, ICs were included into our study design as they represent the highest prevalence in the general population [2] and therefore could serve as kind of a ‘control group’ between ECs and LCs. Most previous studies had neglected this important group, so failed to compare moderate/extreme chronotype behavioural performance and brain processing patterns to the ‘general population’. Chronotype-specificity of semantic processing as an essential cognitive function would lead to significant consequences. A better understanding of the underlying neuronal mechanisms and how semantic knowledge is modulated in different chronotypes will change the way we schedule patient evaluations and importantly, recruitment regimes for participants in experimental research. At the very least, “chronotype” could be included as a covariate, thus added as a factor in data analysis. Otherwise, averaging across subjects could unintentionally hide effects of interest (cf. recent study elaborating especially on this point [29]).

Three specific hypotheses were formulated based on the previous literature. Behaviourally, it was hypothesized that ECs demonstrate slower reaction times than LCs during their subjective evening. Second, we hypothesized that ECs exhibit attenuated activation in brain areas involved in semantic priming performance compared to LCs, e.g. SFG and postcentral gyrus. Third, attenuated brain activation was expected to be present in EC’s hypothalamic area as this group has been reported to be more vulnerable to accumulated sleep pressure.

## Materials and Methods

### Participants

Sixteen healthy, male ECs, 16 LCs and 16 ICs participated in this study. Subjects filled in the Munich Chronotype Questionnaire (Roenneberg et al., 2003), MCTQ, to determine their individual chronotype out of seven potential types: extreme early, moderate early, light early, intermediate, light late, moderate late, extreme late. The present study included ICs, moderate ECs, and extreme (n = 7) to moderate (n = 9) LCs. The MCTQ is freely available at https://www.bioinfo.mpg.de/mctq is a standardized self-rating scale and assesses individual’s phases of entrainment on work and work-free days respectively. In addition, data on primary sleep times, such as bed- and rise-times, clock times of becoming fully awake as well as sleep latency, inertia and daylight exposure are collected. Subjects of the present study filled out the MCTQ in a printed version and afterwards, the principal investigators used the online version of the MCTQ to classify the subjects. The processed algorithms calculate the midsleep on workdays, corrected for midsleep on free days. The MCTQ’s validity is proven via sleep logs, objective measures of activity and rest and physiological parameters. Thus, the MCTQ offers a major tool for chronotype classification. Subjects had an average age of 25.23 years (SD = 5.35) and were recruited by internet alerts, newsletters and flyers and were financially compensated for participation. Study-specific inclusion criteria were: (1) age 18–35; (2) normal or corrected-to-normal vision; (3) right-handedness according to the Edinburgh Inventory of Handedness [30]; and (4) mother tongue German. Besides common fMRI exclusion criteria (e.g. incorporated metal such as a retainer, pacemaker, tattoo etc.), the following also served as exclusion criteria: (1) current or past psychiatric, neurological, or relevant medical disease (e.g. head trauma with unconsciousness > 5 min); (2) daily consumption of more than five cups of coffee or caffeinated drinks; (3) history of night work or shift work; (4) crossing of more than two time zones during the last three months prior to the study; (5) symptoms of a possible sleep disorder according to the Pittsburgh Sleep Quality Inventory (PSQI[31]) or the Epworth Sleepiness Scale (ESS[32]); and (6) a depressed state according to the Beck Depression Inventory (BDI[33]).

The local institutional review board (IRB; Rheinisch-Westfälische-Technische Hochschule Aachen University, Germany, EK 10/157) approved the study protocol, screening questionnaires and consent forms. All subjects provided written informed consent prior to participation. Participants were instructed that the study was aimed at investigating cognitive performance differences in different chronotypes and were requested to maintain a regular sleep-wake schedule the last week prior to admission. Moreover, on the day of the fMRI scan, participants were asked to not practice sports before the scan, to abstain from alcohol and energy drinks and to not consume coffee/tea or caffeinated beverages after 1 p.m. Immediately preceding the fMRI session, each participant rated his/her subjective sleepiness on the Karolinska Sleepiness Scale (KSS[34]) and handed in their sleep diary giving information about the sleep wake behaviour during the last two days prior to the measurement (i.e. bed time, clock time falling asleep, clock time waking up, clock time getting up).

### Stimuli and design

**Fig 1:**
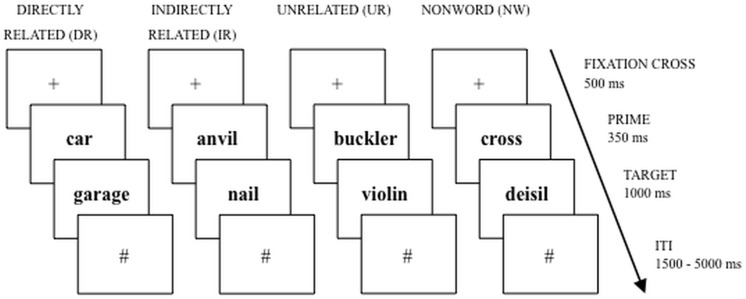
Schematic display of the semantic priming task (Directly related, DR; Indirectly related, IR; Unrelated, UR; Nonword, NW). After presenting a fixation cross for 500ms, the prime word was presented, lasting for 350ms. Immediately after the prime, the target appeared for 1000ms, followed by a hash symbol that was shown for the jittered range of 1500ms to 5000ms (ITI, Inter-trial-interval).

The semantic priming task (Fig 1) based on Sass et al. [12] was implemented in a fast event-related fMRI design. Prime and target words were visually presented (prime: 350 ms, target: 1000 ms) to the participants. Prime words were concrete German words (e.g. “garage”), while target words were either existing concrete German words or a senseless combination of letters (e.g. “maum”). Primes and targets belonged to the same overall conceptual domain (all words depicted only objects), were concrete and imaginable. All stimuli (i.e. primes and targets) matched the criteria of lexical frequency (CELEX database[35]), word length and syllables. An independent pre-test had been conducted to ensure that the DR word pairs had a strong relationship [14]. Twelve volunteers were asked to rate 160 word pair relations on a scale from 1 (= unrelated) to 7 (= highly related). The volunteers were instructed to rate the target words regarding their contextual relatedness and their interaction in time and space. Words selected had to be values of 5 or higher in the pre-test. Trials began with an attention cue “+” (of 500 ms duration) which was followed by the prime (350 ms). Directly after the prime, the target appeared (1000 ms), which was followed by a hash symbol that was shown for the jittered range of 1.5 s to 5 s, see Fig 1.

A total of 180 prime-target pairs were presented in two experimental runs (comprising 90 word pairs, lasting 7 minutes each). Four categories of word pairs existed: Target and prime were either directly related (prime and an associatively related target, e.g. “car–garage”, n = 15 pairs, called “DR”), indirectly related (with the prime being indirectly related to a non-presented mediator, e.g. prime “anvil”–mediator “hammer” *(not shown)–*target “nail”, n = 15, “IR”), or unrelated (“car–frame”, n = 30, “UR”). A pre-test had been conducted to ensure that the DR word pairs had a strong relationship and stimuli for the IR and UR pairs have been validated [14]. Lastly, 30 targets were non-words (e.g. “car–maum”, n = 30, “NW”). NWs were all pronounceable German ‘words’ that were constructed by changing one or two consonants in real world target words.

Participants were required to read both, prime and target, and had to determine whether the target was a real word or a NW by pressing one of two buttons with the left index finger (real word) or middle finger (NW). Left button presses were chosen over right presses to avoid dominant motor activation clusters close to language-prone areas. Reaction times exceeding 3 s and non-responses were classified as errors. Participants were given no information about the construction and arrangement of stimuli and conditions. A brief training run was presented to attain familiarity with the speed of the presented stimuli. The stimuli display was controlled using a Presentation script file (Version 14.8, software package, Neurobehavioral Systems, http://neurobs.com).

### Data acquisition

The fMRI session was conducted ten to twelve hours after each individual's wake up time that was determined by the MCTQ. Scanning was performed on a 3 Tesla scanner (Siemens, Erlangen, Germany) using standard gradients and a 12-channel phase array head coil. Participants lay in a supine position. Head movement was limited by foam padding within the head coil. To ensure optimal visual acuity, participants were offered MRI-compatible glasses if necessary.

The experimental runs comprised 223 whole-brain echo planar imaging (EPI) scans. These were preceded by three initial dummy scans allowing for signal saturation effects. Thirty-two slices (3 mm thickness, distance factor 40%) were positioned parallel to the AC/PC line. The following parameters were applied: matrix size 64 x 64; field-of-view (FOV), 200 mm x 200 mm; echo time (TE), 30 s; repetition time (TR), 1.94 s. For anatomical localization, a magnetization-prepared rapid gradient echo (MP-RAGE) sequence was acquired during the same imaging session (TR = 2250 ms; TE = 3.03 ms; ST = 1 mm; FOV = 256 x 256 mm; voxel size = 1.0 x 1.0 x 1.0 mm).

### Behavioural data analysis

Statistical analysis was performed using SPSS (IBM SPSS Statistics 19; http://www.spss.com). Reaction time was measured from the moment the target was presented until the participant made a response. Reaction times and percent correct responses served as dependent variables. The nonword (NW) condition is often excluded in analyses of semantic priming (behavioural and fMRI) data in typical linguistic experiments but in order to i) include all variables that are part of our experimental design, ii) satisfy readers from all fields involved (linguistics, chronobiology, neuroimaging, cognitive neuroscience) while iii) focussing on chronobiology as well as semantic processing, we provide results of two analysis approaches, namely a restricted 3x3 (group: LC, EC, IC) by condition (“prime-target relation”: DR, IR, UR, excluding NW) ANOVA and a full 3x4 ANOVA (group by condition DR, IR, UR, NW) for investigating chronotype-specificities. For both ANOVAs, the “prime-target relation” served as dependent variables and “chronotype” as a between subject factor. Planned *t*-tests were conducted to clarify potential sources interactions between relatedness. Kolmogorov–Smirnov tests confirmed the normal distribution of the data (all P*s* > 0.5). Demographic data (i.e. age, education), individual sleep preferences (i.e. KSS, ESS, PSQI, sleep diary entries) and lifestyle habits (i.e. nicotine and alcohol consumption, BDI) were analysed using one-way ANOVA with “chronotype” as between–subject factor (post-hoc test, Bonferroni–corrected).

### fMRI data analysis

Prescans were discarded prior to analysis. The FEAT (FMRI Expert Analysis Tool) module of the FMRIB Software Library (FSL, http://www.fmrib.ox.ac.uk/fsl) was used for image processing and statistical analyses. Motion correction was performed using MCFLIRT [36], removal of non-brain structures from the EPI volumes by BET [37], spatial smoothing was performed with a Gaussian kernel of 5 mm FWHM and high-pass temporal filtering. Functional scans were registered to the MNI152 standard space (FLIRT [38]). GLM time-series statistical analysis of individual data sets was carried out using FILM with local autocorrelation correction [39]. Explanatory variables (EV) were created for each stimulus type (DR, UR, IR and NW), using the stimulus onset times as event markers. Each EV was convolved with a double-gamma haemodynamic response function (HRF). The first level (single subject) analysis yielded statistical maps representing the mean response in each stimulus category. A second level fixed effect analysis was performed for each subject individually to obtain contrast estimates for each of the following comparisons: the unrelated condition was subtracted from the related conditions (DR > UR; IR > UR; DR < UR; IR < UR). Additionally, the IR condition was subtracted from the DR condition (DR > IR; DR < IR). Moreover, the NW condition was subtracted from the UR, DR and IR condition (UR > NW; DR > NW; IR > NW; UR < NW; DR < NW; IR < NW). Higher-level analysis (group level mixed effects analyses) was carried out with FLAME (FMRIB’s Local Analysis of Mixed Effects, [17], Z > 2.3, P = .01, cluster corrected). In FSL, if cluster correction thresholding is selected, a Z statistic threshold is used to define the number of contiguous clusters. Then each cluster's estimated significance level is compared with the cluster probability threshold. Significant clusters are then used to mask the original Z statistic image for later production of colour blobs. Functional data were imported to MRIcron [40] for display purposes. The percentage signal change was extracted from regions of interest (ROIs) using the Featquery module of FSL. The ROIs were defined as the peak voxel in each activated region for the main effect chronotype. A *brain-behaviour analysis*, i.e. a bivariate correlation analysis (Pearson, *r*) of the percentage signal change and self-reported demographic, sleep characteristics and lifestyle habits with brain activation for ECs, ICs, and LC was performed.

## Results

### Demographic, sleep and lifestyle characteristics

Detailed, group-specific demographic, sleep and lifestyle characteristics are shown in Table 1. Subjects had an average age of 25.23 years (SD = 5.35). One EC, two ICs and eleven LCs reported regular use of nicotine. Eight individuals reported to refrain from any alcohol consumption (five ECs and three LCs). LCs reported higher current daily nicotine consumption compared to ECs and ICs (post-hoc test, Bonferroni corrected, P = .02, P = .021, respectively) and consuming more units of alcoholic beverages per week (post-hoc test, Bonferroni corrected P = .001, P = .004, respectively). No significant differences were detected for age, education, symptoms of a possible sleep disorder according to the PSQI, increased daytime sleepiness according to the ESS, subjective sleepiness according to the KSS and self-rated depressive mood according to the BDI scores. Analysis of sleep diary entries (i.e. bed time, clock time falling asleep, clock time waking up, clock time getting up) for the last two sleep/wake cycles prior to measurement revealed significant chronotype differences between LCs and ECs in so far as the LCs show a delayed sleep/wake cycle as compared to the ECs (Bonferroni corrected). For the waking up time, two days before the measurement, an additional significant group effect for the ICs (i.e. delayed sleep/wake cycle) as compared to ECs was reached (Bonferroni corrected).

**Table 1:** Demographic and sleep characteristics, self-ratings (mean, standard deviation (SD)) of early (EC), intermediate (IC) and late chronotypes (LC).

	EC	IC	LC	Directionality
**Age**	26.88 (6.41)	23.75 (4.29)	23.06 (2.91)	
**Education** ^**a**^	16.53 (2.5)	16.13 (2.75)	15.25 (2.9)	
**PSQI**	3.88 (2.06)	3.19 (1.11)	4.75 (1.88)	
**ESS**	7.38 (3.72)	8.94 (3.2)	7.00 (2.28)	
**KSS**	4.20 (2.0)	4.67 (1.59)	4.81 (1.47)	
**BDI**	3.00 (3.58)	3.81 (3.62)	5.53 (5.48)	
**Smoking** ^**b**^	0.54 (2.15)	0.44 (1.78)	3.96 (5.21)	LC > EC^*^, LC > IC^*^
**Alcohol** ^**c**^	1.96 (2.2)	4.10 (3.17)	9.69 (8.53)	LC > EC^**^, LC > IC^**^
**Bed time** ^**d**^	22:54 (01:17)	23:35 (01:31)	00:14 (01:26)	LC > EC^*^
**Time falling asleep** ^**d**^	23:04 (01:16)	00:03 (01:36)	00:36 (01:23)	LC > EC^*^
**Time waking up** ^**d**^	05:43 (01:00)	07:03 (01:02)	07:32 (01:54)	LC > EC^**^, IC > EC^*^
**Time getting up** ^**d**^	05:54 (00:57)	07:19 (01:17)	07:53 (02:16)	LC > EC^**^
**Bed time** ^**e**^	23:05 (01:28)	23:27 (00:54)	00:28 (02:02)	LC > EC^*^
**Time falling asleep** ^**e**^	23:20 (01:35)	23:59 (00:47)	00:49 (01:55)	LC > EC^*^
**Time waking up** ^**e**^	05:52 (00:48)	07:00 (01:03)	07:50 (01:42)	LC > EC^**^
**Time getting up** ^**e**^	06:13 (01:01)	07:16 (01:12)	08:05 (01:39)	LC > EC^**^

Abbreviations:

PSQI, Pittsburgh Sleep Quality Inventory

ESS, Epworth Sleepiness Scale

KSS, Karolinska Sleepiness Scale

BDI, Beck Depression Inventory.

*P < .05

** P < .01

^**a**^ expressed in number of school and study years.

^**b**^ expressed in typical number of cigarettes per day.

^**c**^ expressed in typical number of standard alcoholic drinks per week.

^**d**^ sleep/wake cycle two days before the measurement day.

^**e**^ sleep/wake cycle one day before the measurement day.

### Behavioural data

The 3 x 3 ANOVA revealed a significant main effect of “chronotype” (i.e. ECs, ICs, LCs), F_(2,45)_ = 3.18, P < .05, with ECs responding significantly slower than LCs (P < .05). No interaction between “chronotype” and “prime-target relation” could be revealed. Similarly, the 3 x 4 ANOVA revealed a significant main effect of “chronotype” (i.e. ECs, ICs, LCs), F_(2,45)_ = 3.55, P < .05, with ECs responding significantly slower than LCs (P < .05). No interaction between “chronotype” and “prime-target relation” could be revealed.

Additional post-hoc analysis of ONEWAY ANOVA with “prime-target relation” as dependent variables and “chronotype” as factor revealed that LCs responded significantly faster than ECs during IR, UR and NW stimuli (post-hoc analysis, P < .05, see Table 2).

**Fig 2:**
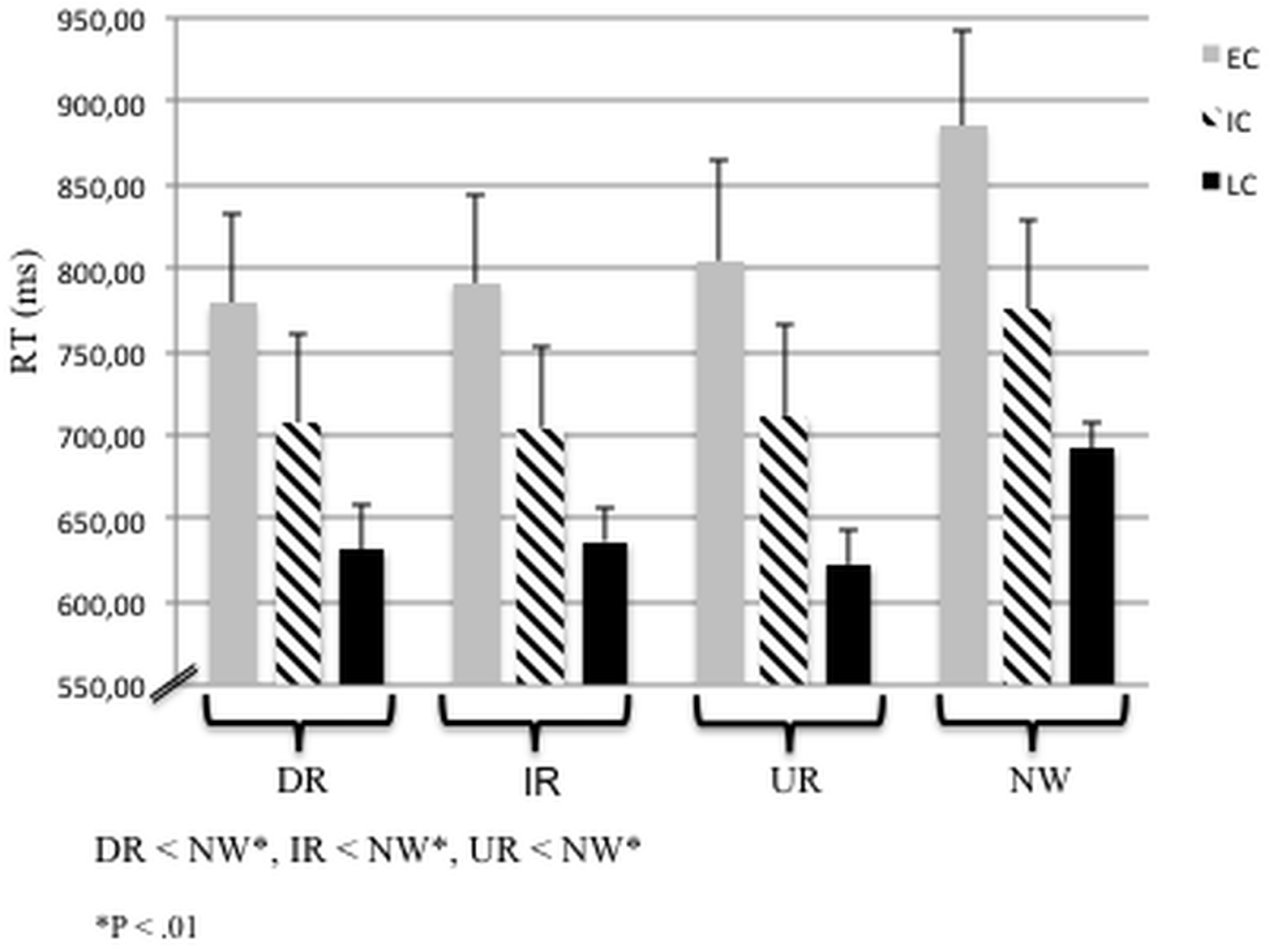
Subjects’ Performance during the fMRI Semantic Priming Task. Presented are mean values of reaction times (RT, ms) of directly related (DR), indirectly related (IR) and unrelated (UR) prime-target word pairs versus non-words (NW) plus SE (Standard Error). Asteriks mark significant differences between the RTs of the semantic priming conditions (P < .01).

Paired t-tests confirmed that subjects, independently of chronotype, processed the semantic priming conditions DR, IR and UR faster than NW (all P < .01, see Fig 2).

**Table 2:** Mean reaction times of early (EC), intermediate (IC) and late chronotypes (LC) and standard error (SE) in ms. Directionality refers to posthoc group comparisons (one-way ANOVA, Bonferroni corrected).

	EC	IC	LC	Directionality
DR	778.44 (54.6)	706.56 (52.9)	631.31 (25.9)	
IR	790.94 (53.03)	704.19 (48.6)	636.19 (20.6)	LC < EC*, P = .045
UR	804.38 (59.67)	710.31 (54.9)	622.31 (20.8)	LC < EC*, P = .032
NW	884.94 (57.7)	775.25 (53.5)	692.00 (15.2)	LC < EC*, P = .015

Abbreviations:

DR, directly related

IR, indirectly related

UR, unrelated

NW, nonwords.

* P < .05

Subjects responded to 96.51% of the items correctly; therefore incorrect answers were excluded from further analyses. A main effect of “chronotype” for accuracy could not be revealed (F_(2,45)_ = 1.76, P = .19). Moreover, neither a main effect of “prime-target relation” (all paired t-tests n.s.) nor an interaction between “chronotype” and “prime-target relation” could be reached for accuracy (F_(6,135)_ = 1.21, P = .31).

### Imaging data

#### Chronotype

**Fig 3:**
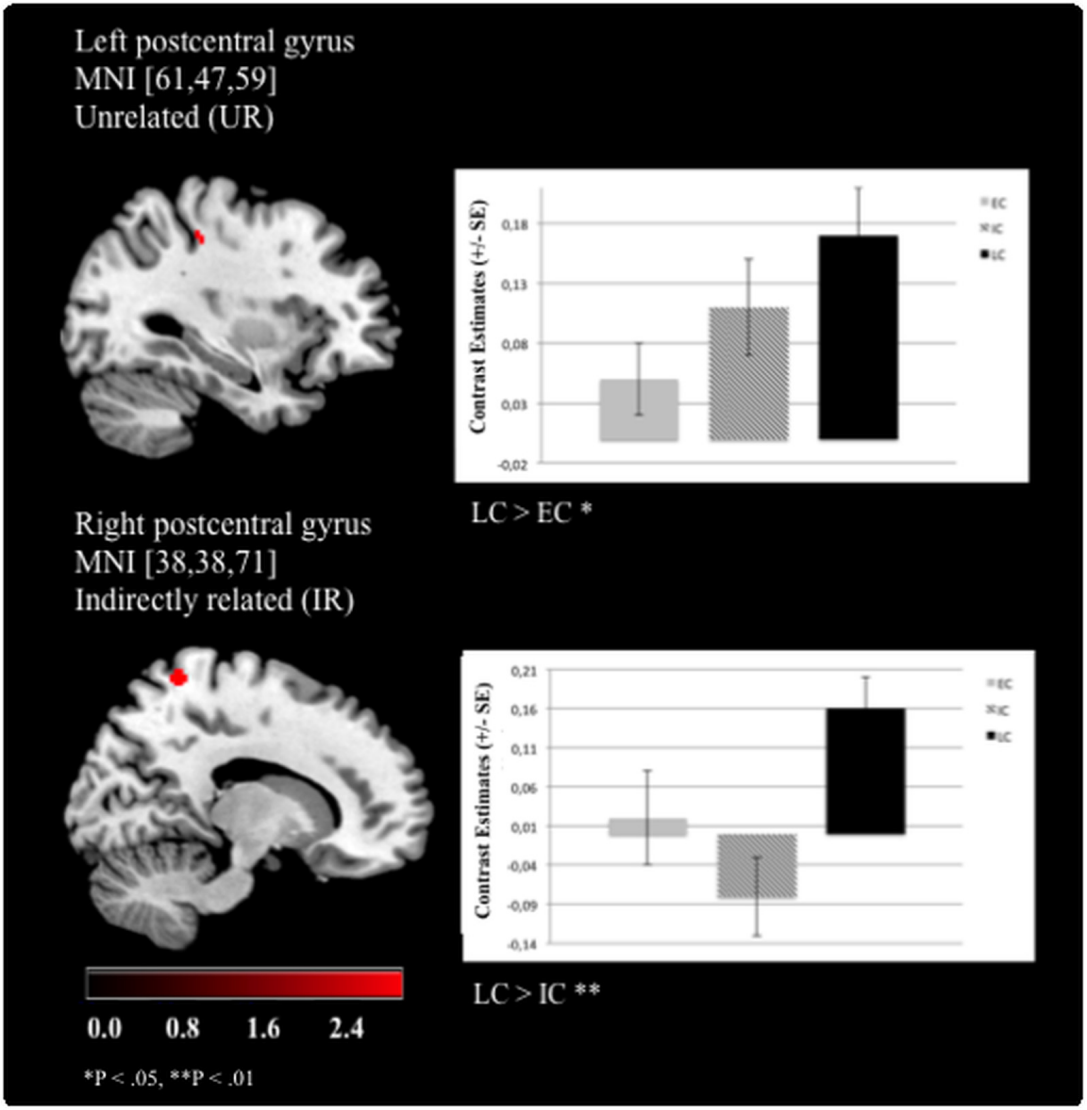
Late chronotypes show enhanced BOLD activation in the postcentral gyri. For the left postcentral gyrus, late chronotypes (LC) showed enhanced brain activation when processing unrelated (UR) prime-target word pairs compared to early chronotypes (EC). For the right postcentral gyrus, LCs exhibited a higher BOLD response than intermediate chronotypes (IC) when processing indirectly (IR) related prime-target words (Functional Imaging Data: MNI [x, y, z], mixed effects FLAME, P < .01).

**Fig 4:**
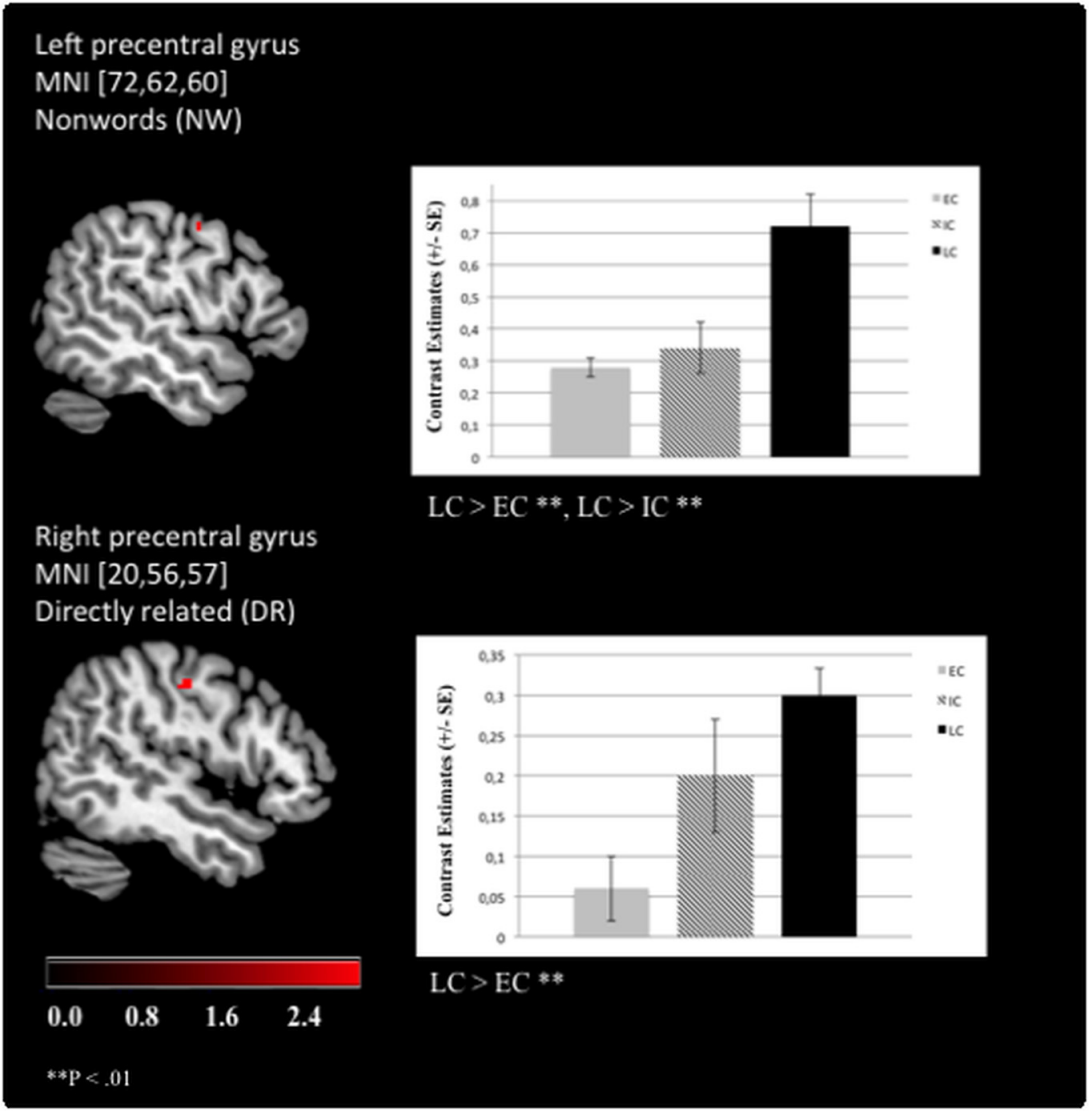
Late chronotypes show enhanced BOLD activation in the precentral gyri. For the left precentral gyrus, late chronotypes (LC) showed enhanced brain activation as compared to early (EC) and to intermediate (IC) chronotypes when processing non-words (NW). For the right precentral gyrus, LCs exhibited a higher BOLD response than ECs when processing directly (DR) related prime-target words (Functional Imaging Data: MNI [x, y, z], mixed effects FLAME, P < .01).

**Fig 5:**
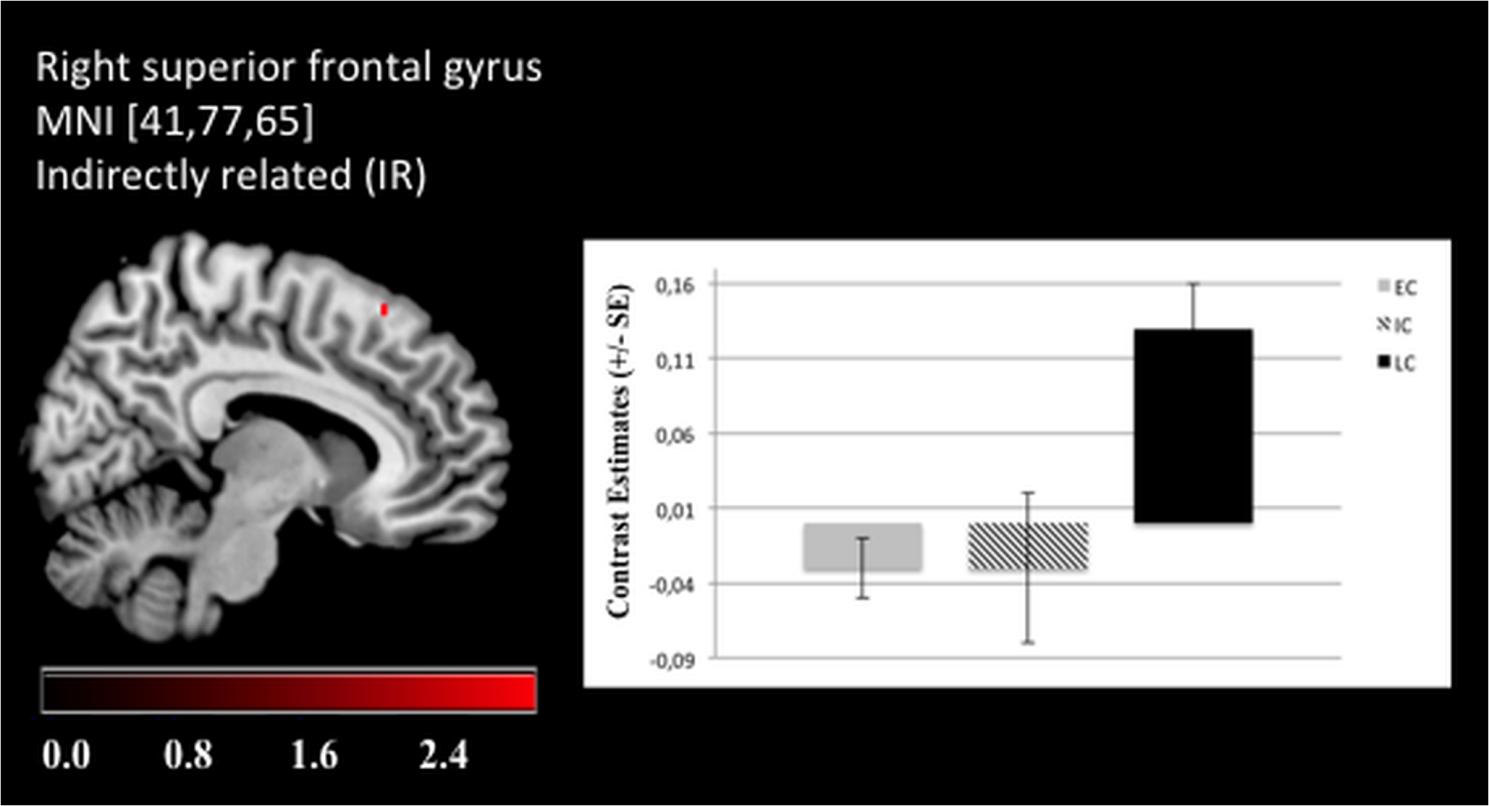
Late chronotypes show enhanced BOLD activation in the superior frontal gyrus. For the right superior frontal gyrus, late chronotypes (LC) showed enhanced brain activation when processing indirectly (IR) related prime-target word pairs compared to early (EC) chronotypes (Functional Imaging Data: MNI [x, y, z], mixed effects FLAME, P < .01).

**Fig 6:**
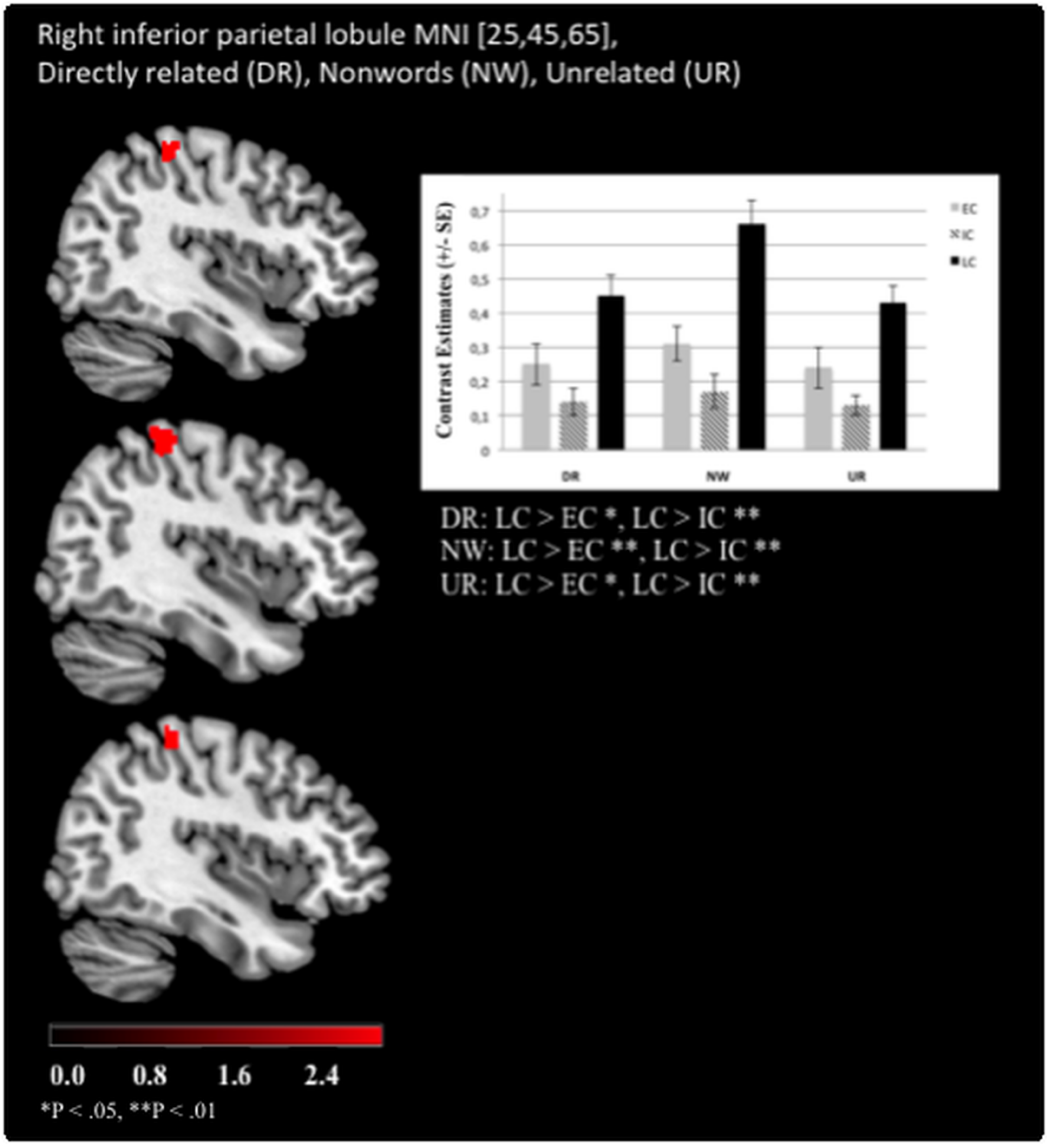
Late chronotypes show enhanced BOLD activation in the inferior parietal lobule. For the right inferior parietal lobule, late chronotypes (LC) showed enhanced brain activation when processing directly (DR) and unrelated (UR) prime-target word pairs as well as processing non-words (NW) compared to early (EC) and intermediate (IC) chronotypes (Functional Imaging Data: MNI [x, y, z], mixed effects FLAME, P < .01).

The significant effects of “chronotype” are summarized in Table 3. Overall, LC showed stronger activation patterns than EC or IC. Specifically, enhanced left postcentral gyrus (Brodmann area (BA) 3) activation was revealed for LCs as compared to ECs when UR word pairs were presented (Fig 3). Moreover, LCs exhibited stronger right postcentral gyrus (BA 7) activation than ICs during IR pairs (Fig 3). LCs were also characterized by increased left precentral gyrus (BA 4) activation compared to ECs when processing NWs and for the right precentral gyrus (BA 4) in processing DR word pairs (Fig 4). Additionally, enhanced activation of the right SFG (BA 6) was revealed compared to ECs (Fig 5). Furthermore, LCs showed stronger right inferior parietal lobule activation (BA 40) than ICs in processing of DR, UR and NWs (Fig 6). The following contrasts were analysed for group differences but effects of “chronotype” did not reach statistical significance: the unrelated condition was subtracted from the related conditions (DR > UR; IR > UR; DR < UR; IR < UR). Additionally, the IR condition was subtracted from the DR condition (DR > IR; DR < IR). Moreover, the NW condition was subtracted from the UR, DR and IR condition (UR > NW; DR > NW; IR > NW; UR < NW; DR < NW; IR < NW).

**Table 3:** Functional Imaging data. Effects of chronotype. Mixed effects FLAME, P < .01.

Semantic Priming Condition	x^a^	y^a^	z^a^	Z-max	Cluster Index	Correspondent anatomical location	Directionality
DR	20	56	57	3.5	1	R, precentral gyrus (BA^b^4)	LC > EC^*^
	25	45	64	3.6	2	R, inferior parietal lobule (BA40)	LC > IC^*^
IR	41	77	65	3.3	1	R, superior frontal gyrus (BA6)	LC > EC^*^
	38	38	71	3.5	2	R, postcentral gyrus (BA7)	LC > IC^*^
NW	72	62	60	3.3	1	L, precentral gyrus (BA4)	LC > EC^*^
	25	46	66	4.1	2	R, inferior parietal lobule (BA40)	LC > IC^*^
UR	61	47	59	3.4	1	L, postcentral gyrus (BA3)	LC > EC^*^
	25	45	65	3.8	2	R, inferior parietal lobule (BA40)	LC > IC^*^

Abbreviations:

DR, directly related

IR, indirectly related

UR, unrelated

NW, non-words.

^a^MNI coordinates.

^b^Brodmann area.

R = Right Cerebrum.

L = Left Cerebrum.

^*^ P < .01.

### Prime-target relation

Processing of “prime-target relation” activated a bilateral network of task-related brain regions (see S1 Table). Across all chronotypes, directly linked prime-target words activated left-lateralized superior frontal brain areas while indirectly related words revealed bilateral activation of the paracingulate gyrus. Unrelated prime-target words led to bilateral activation mainly in the paracingulate gyrus and in the left superior frontal gyrus. Moreover, the left superior frontal gyrus was active during the processing of NWs.

### Brain-behaviour analysis

Bivariate correlation analysis of self-reported demographic, sleep characteristics and lifestyle habits with brain activation (based on contrast estimates, main effect “chronotype”) of ECs, ICs, and LC did not yield significant results (see S2 Table). Bivariate correlation analysis of reaction times (mean, ms) of DR, IR, UR and NWs with significant BOLD activations of ECs, ICs and LCs reached a negative association between the activation of the right postcentral gyrus (BA 7) and the reactions times during IR processing for ICs (*r* = -.8, P < .01; see S3 Table).

## Discussion

The present study aimed at investigating whether different chronotypes show distinct behavioural and neural processing of semantic stimuli. In particular, we tested whether ECs, LCs and ICs differ on a semantic priming task. Confirming our first hypothesis, ECs exhibited significant slower reaction times than LCs. In line with our second hypothesis, ECs showed attenuated BOLD responses in brain areas relevant to semantic priming, namely the pre- and postcentral gyri and the SFG. Contradicting our third hypothesis, ECs did not exhibit decreased hypothalamic activation during their subjective evening.

Chronotype-specificity was revealed with ECs showing significant slower reaction times than LCs (RTs of IR and UR stimuli as well as NWs). It is important to note that all three experimental groups had been tested at 10–12 hours after their individual wake up times, thus at different times during the late afternoon/early evening. This approach guaranteed that ECs, LCs and ICs were equally entrained [2]. Our present results support previous studies that exhibited significantly lower objective vigilance (as measured by the PVT) and slower reactions times of executive functions (as measured by the Stroop-task) for ECs as compared to LCs during the subjective evening hours, although the amount of prior wakefulness and circadian entrainment was the same for both chronotype groups [41]. Along these behavioural findings, Schmidt and colleagues showed that ECs reached higher electroencephalography (EEG) slow-wave activity (SWA) values during the night after the fMRI scan than LCs during the first non-rapid eye movement (NREM) sleep episode, despite the same amount of time previously spent awake, suggesting higher levels of accumulated sleep pressure. It was concluded that EC individuals experience a higher vulnerability to increasing time spent awake across a normal waking day. Indeed, inter-individual differences in vulnerability to increasing time spent awake had been shown to cluster around different behavioural dimensions, namely cognitive processing ability and behavioural alertness [41, 42]. However, in our present study, EC, IC and LC did not differ in their subjective sleepiness as assessed by the KSS, suggesting that subjects felt equally entrained (i.e. awake). Accordingly, our results of slower reaction times in EC are not related to differences of subjects’ individual experience of sleepiness upon testing, but suggest an inherent chronotype-specific disposition for the processing of linguistic semantic processing. Hence, our results speak for a circadian and sleep-wake homeostatic influence on some higher cognitive functioning. Future research in the field of chronobiology will have to demonstrate whether these findings extend to other linguistic–or even broader, to other neuropsychological–functions (e.g. verbal fluency, verbal memory, but also working memory, decision making etc.) or are limited to semantic priming effects.

Confirming our second hypothesis, ECs showed attenuated BOLD responses in several task-relevant brain areas: a) in the left postcentral gyrus for the processing of UR word pairs, b) in the left and right precentral gyrus for NWs and DR words, respectively, and c) in the right superior frontal gyrus for IR words. More specifically, LCs showed enhanced BOLD activation in the left postcentral gyrus (BA 3) accompanied by faster reaction times during the processing of unrelated word pairs compared to ECs. Besides clear evidence for the somatosensoric representation of the human body [43], neural activation in this region has been linked to healthy subjects’ increased reading ability (i.e. performance on a sentence comprehension task [28]). The present finding of enhanced postcentral activation and faster reaction times suggests that LCs may have realized much faster than ECs that the target word was a real word, although it was semantically unrelated to the prime. An unrelated word pair consists of words that do not trigger associations between the prime and the target word, i.e. no priming effect occurs. Therefore, unrelated words usually reflect longer reaction times than related word pairs [14]. To conclude, our finding suggests that LCs could activate this task-relevant area involved in the reading ability during lexical decisions more effectively. In addition, ECs exhibited attenuated BOLD activation in the left precentral gyrus, which has been primarily implicated in motor function [44] but has also been conceptualized also to encoding processes during reading [22]. Thus, we hypothesize that LCs might have classified (i.e. encoded) pronounceable non-words more efficiently than ECs, resulting successfully in faster reaction times in deciding that a non-word has been presented. Moreover, recent research showed that activation of the right precentral gyrus is frequently reported during language tasks (cf. meta-analysis [45]). It is still under debate whether the right precentral gyrus could be especially related to motor processes that are involved in language processing. Considering that our (right-handed) subjects used the left fingers for the button presses (to avoid dominant motor activation clusters close to language-prone areas), the lexical decision task in the present study involves a motor response that is combined with semantic processing. In a previous study probing a totally unrelated task in a clinical population, we argued that increased precentral activation may reflect the organism’s preparation for actions [46]. We observed enhanced BOLD activation within this area for LCs as compared to ECs when processing DR word pairs. These word pairs usually exhibit the strongest priming effect (i.e. compared to indirectly related and unrelated word pairs) because the triggered associations between these words are directly connected. The correlation analysis that focused on the relationship between the reaction times during the processing of DR word pairs and the significant contrast estimates of the BOLD activation in the right precentral gyrus (cf. S3 Table) for LCs and ECs did not reach significance, suggesting that brain activation was not associated with faster reaction times for LCs as compared to ECs. Moreover, although we cannot fully rule out the motor component, the activation was not found in the superior parts of the precentral gyrus, which may be close to the hand area (cf. Fig 4). In processing indirectly related word pairs, ECs showed diminished BOLD responses in the right superior frontal gyrus (i.e. BA 6) as compared to LCs. BA 6 is involved in the planning of coordinated movements [43] but activation clusters in this area were associated with the recognition of semantic relationships between words, too [25, 34]. Moreover, ECs responded significantly more slowly to indirectly related words than LCs. Importantly, IR word pairs are associated indirectly via a non-presented mediator, therefore representing a stronger associative relationship than the unrelated but a weaker association than the directly related word pairs. This observation was verified in the present study with a stable significant semantic priming contrast across the chronotype groups: IR word pairs led to a stronger activation of the superior frontal and the paracingulate gryrus as compared to the DR word pairs (cf. S1 Table). Concerning chronotype-specificity, the results demonstrate that LCs reached significantly faster reaction times during the processing of indirectly related word pairs than ECs (see Table 2).

We also provide evidence for attenuated BOLD responses for ICs as compared to LCs in the right inferior parietal lobule (BA 40) for DR, UR word pairs and NWs. In more detail, activations in this region were reported to reflect subjects’ ability to efficiently maintain attention to the current linguistic as well as non-linguistic goals (see review by [47]). LCs showed enhanced BOLD activation in this region, therefore showing less difficulties maintaining sustained attention to the lexical decision task. On the contrary, for IR word pairs, attenuated BOLD responses for ICs as compared to LCs were exhibited in the right postcentral gyrus (BA 7). BA 7 has been linked to higher-level processing tasks, including the activation of association during language tasks [48]. In addition, brain-behavior analysis revealed that the attenuated BOLD response of the ICs is negatively correlated with higher reaction times (see S3 Table).

Previous studies using “free-running” subjects argued that their findings (i.e. ECs showing slower reaction times and attenuated BOLD responses in task-relevant brain areas as compared to LCs) stem from the fact that ECs are more vulnerable to the homeostatic sleep pressure that accumulates during the normal waking day [49]. Results were affirmed by showing diminished hypothalamic activation and significant higher subjective sleepiness values (assessed by the Karolinska Sleepiness Scale) for ECs during their subjective evening as compared to LCs. Contrary to these results, and to our third hypothesis, we could not replicate these findings with our current results: LCs did not present increased hypothalamic activation nor did they feel less sleepy than ECs (and ICs). This seeming discrepancy between our findings and previous reports may in part be related to the sample composition: Contrary to previous studies, we included moderate not extreme ECs (according to the MCTQ) in the current study as they are represented with a higher prevalence especially in the age group under investigation [2]. Nevertheless, analysis of sleep diary entries (i.e. bed time, clock time falling asleep, clock time waking up, clock time getting up) for the last two sleep/wake cycles prior to measurement revealed significant chronotype differences between LCs and ECs in so far as the LCs show a delayed sleep/wake cycle as compared to the ECs. Thus, although our subjects kept their usual working hours, they are entrained according to their individual chronotype. Importantly, our results now show that even moderate, not “free-running” chronotypes present characteristic brain activation patterns.

The current study design successfully ruled out that modulating factors, e.g. sleep disturbances, affected our results. Subjects neither suffered from sleep disturbances according to well-established self-rating questionnaires nor reported symptoms of a depressive mood (see Table 1). A thorough semi-structured screening procedure confirmed that self-reported consumption of numbers of alcoholic drinks per week and number of cigarettes per day in the present study did not qualify for alcohol or nicotine abuse. Nevertheless, while having excluded subjects with excessive use of nicotine and alcohol, consumption of alcohol and nicotine was more prominent in LCs as compared to ICs and ECs. It is well-described that LCs consume more alcohol and nicotine than ECs [50, 51]: it is argued that LCs exhibit a chronic form of functional jet jag [52] because their endogenous sleep-/wake rhythms rarely fit conventional social schedules (e.g. working hours, school). However, secondary statistical analyses revealed no significant correlations between alcohol consumption and contrast estimates of the significant activated brain areas (see S2 Table) showing that alcohol consumption is not related to our findings. While nearly all of our participants consumed alcohol, smokers among the chronotype groups were unequally distributed with only one and two smokers among the ECs and ICs, respectively, but eleven LC smokers. Likewise, correlation analysis confirmed that smoking is not related to our findings.

The following limitations should be highlighted. First, we only included male subjects to eliminate potential hormonal effects and to avoid adding another level of statistical complexity. Hence, our results speak for males only and need to be replicated in females. Second, the assessment of melatonin and cortisol levels as well as the use of actimeters® for verifying the entrainment of the subjects was not part of the current study protocol. However, the fMRI session was conducted ten to twelve hours after each individual’s wake up time that was determined by the MCTQ (i.e. ensuring comparable entrainment). Third, our subjects kept their usual work hours and were therefore not “free-running” (i.e. adjusted to an artificial cycle). Thus, entrainment could be masked by social duties (e.g. working hours, family etc.).

To summarize, the present study for the first time examined chronotype-specific brain activation during semantic processing. We show that individuals with specific chronotypes differentially recruit task-relevant brain areas. Our results have implications for instance for shift work and fixed working hours arise, as employees may be forced to perform complex tasks at times where their individual chronotype, sleep pressure and associated brain function may negatively impact on performance. Our findings also have implications for fMRI studies in cognitive neuroscience: while we particularly aimed at probing potential chronotype-specific differences, we might have included the very same participants as healthy normal controls into any other experimental study that will increase statistical variance.

Consequently, we suggest that participants might be tested according to their individual chronotype, i.e. investigating all participants in their optimal times of the day when there are equally entrained. At the very least, “chronotype” could be included as a covariate, thus added as a factor in data analysis. Otherwise, averaging across subjects could unintentionally hide effects of interest. Moreover, future studies should include examination of gene expression as previous studies associated the vulnerability to sleep loss with the polymorphism in the PER3 clock gene [53]. In addition, to draw a complete picture of chronotype-specificity and the potential underlying associated synchrony effects of brain activation, subjects should be tested in their subjective optimal as well as non-optimal times. Furthermore, further studies have to clarify whether LCs could potentially have a performance advantage, e.g. in attention, not just compared to ECs but also to ICs during their subjective evening. Thus, future studies have to include ICs into their study design.

## Supporting Information

S1 Table MNI coordinates of direct (DR), indirect (IR), unrelated (UR) semantic priming and non-words (NW) BOLD activations and significant semantic priming contrasts (P < .01, cluster corrected) in early (EC), intermediate (IC) and late chronotypes (LC). (DOCX)

S2 Table Bivariate correlation analysis (Pearson, *r*) of self-reported demographic, sleep characteristics and lifestyle habits with anatomical regions of significant BOLD activation (based on contrast estimates) of early (EC), intermediate (IC) and late (LC) chronotypes. Abbreviations: PSQI, Pittsburgh Sleep Quality Inventory; ESS, Epworth Sleepiness Scale; KSS, Karolinska Sleepiness Scale; BDI, Beck Depression Inventory.
(DOCX)

S3 Table Bivariate correlation analysis (Pearson, *r*) of reaction times (RT, mean, ms) of directly (DR), indirectly (IR), unrelated (UR) word pairs and non-words (NW) with anatomical regions of significant BOLD activation (based on Functional Imaging Results, contrast estimates) for early (EC), intermediate (IC) and late (LC) chronotypes. (DOCX)
