# Supplementary material for: Chronotype Modulates Language Processing-Related Cerebral Activity during Functional MRI (fMRI)
Source: PLoS One. 2015 Sep 23;10(9):e0137197. doi: 10.1371/journal.pone.0137197 (PMC4580315; doi:10.1371/journal.pone.0137197)
Supplement: S1 Table — (DOCX) [file pone.0137197.s001.docx]

| **Chronotype** | | **x^a^** | **y^a^** | **z^a^** | **Z-max** | **Cluster Index** | **Correspondent anatomical location** | |
| --- | --- | --- | --- | --- | --- | --- | --- | --- |
| **EC** | |  |  |  |  |  |  | |
| DR | | -6 | 10 | 60 | 5.5 | 1 | L, superior frontal gyrus | |
|  | | -26 | -50 | 44 | 4.9 | 2 | L, superior parietal lobule | |
| IR | | -6 | 8 | 58 | 5.8 | 1 | L, paracingulate gyrus | |
|  | | -28 | -50 | 44 | 5.0 | 2 | L, superior parietal lobule | |
| NW | | -8 | 8 | 48 | 5.9 | 1 | L, suppl. motor cortex | |
| UR | | -6 | 8 | 48 | 5.4 | 1 | L, paracingulate gyrus | |
|  | | -44 | -42 | 48 | 4.6 | 2 | L, supramarginal gyrus | |
| IR > DR | | -6 | 8 | 48 | 5.4 | 1 | L, paracingulate gyrus | |
|  | | -44 | -42 | 48 | 4.6 | 2 | L, inferior parietal lobule | |
| DR > UR; IR > UR; UR > DR; UR > IR; DR > IR; UR > NW; DR > NW; IR > NW; NW > UR; NW > DR; NW > IR | | | | | | | Non-significant | |
| **IC** | |  |  |  |  |  |  | |
| DR | | -2 | 6 | 62 | 5.6 | 1 | L, suppl. motor cortex | |
| IR | | 2 | 16 | 44 | 5.3 | 1 | R, paracingulate gyrus | |
| NW | | -8 | 20 | 50 | 5.4 | 1 | L, superior frontal gyrus | |
|  | | 46 | -26 | 54 | 5.2 | 2 | R, postcentral gyrus | |
| UR | | 2 | 16 | 44 | 5.5 | 1 | R, paracingulate gyrus | |
| DR > NW | | -14 | -88 | -6 | 4.3 | 1 | L, lingual gyrus | |
| IR > NW | | -8 | -90 | -10 | 4.8 | 1 | L, lingual gyrus | |
| IR > DR | | 2 | 16 | 44 | 5.5 | 1 | R, paracingulate gyrus | |
| UR > NW | | -10 | -90 | -8 | 4.0 | 1 | L, lingual gyrus | |
| DR > UR; IR > UR; UR > DR; UR > IR; DR > IR; NW > DR; NW > IR; NW > UR | | | | | | | Non-significant | |
| **LC** | |  |  |  |  |  |  | |
| DR | | 0 | 20 | 50 | 6.1 | 1 | L, superior frontal gyrus | |
|  | | -42 | -32 | 44 | 5.3 | 2 | L, inferior parietal lobule | |
| IR | | -8 | 18 | 46 | 5.7 | 1 | L, paracingulate gyrus | |
| NW | | 0 | 20 | 50 | 6.2 | 1 | L, superior frontal gyrus | |
| UR | | 0 | 20 | 50 | 6.0 | 1 | L, superior frontal gyrus | |
| DR > UR | | -14 | -74 | -28 | 5.6 | 1 | R, cerebellum | |
| IR > DR | | 0 | 20 | 50 | 6.0 | 1 | L, superior frontal gyrus | |
| NW > UR | | 52 | -62 | -6 | 4.5 | 1 | R, middle occipital gyrus | |
|  | | -36 | -42 | -32 | 3.3 | 2 | L, cerebellum | |
| IR > UR; UR > DR; UR > IR; DR > IR; UR > NW; DR > NW; IR > NW; NW > DR; NW > IR | | | | | | | Non-significant | |
|  |  | | | |  | | |  |

**S 1 Table. MNI coordinates of direct (DR), indirect (IR), unrelated (UR) semantic priming and non-words (NW) BOLD activations and significant semantic priming contrasts (P < .01, cluster corrected) in early (EC), intermediate (IC) and late chronotypes (LC)**.

^a^MNI coordinates. L = Left Cerebrum. R = Right Cerebrum
