# Supplementary material for: Chronotype Modulates Language Processing-Related Cerebral Activity during Functional MRI (fMRI)
Source: PLoS One. 2015 Sep 23;10(9):e0137197. doi: 10.1371/journal.pone.0137197 (PMC4580315; doi:10.1371/journal.pone.0137197)
Supplement: S2 Table — Abbreviations: PSQI, Pittsburgh Sleep Quality Inventory; ESS, Epworth Sleepiness Scale; KSS, Karolinska Sleepiness Scale; BDI, Beck Depression Inventory. (DOCX) [file pone.0137197.s002.docx]

**S2 Table**. **Bivariate correlation analysis (Pearson, *r*) of self-reported demographic, sleep characteristics and lifestyle habits with anatomical regions of significant BOLD activation (based on contrast estimates) of early (EC), intermediate (IC) and late (LC) chronotypes.** Abbreviations: PSQI, Pittsburgh Sleep Quality Inventory; ESS, Epworth Sleepiness Scale; KSS, Karolinska Sleepiness Scale; BDI, Beck Depression Inventory.

| **Anatomical region of significant BOLD activation** | **DR**  **R, precentral gyrus**  **LC > EC** | | **DR**  **R, inferior parietal lobule**  **LC > IC** | | **IR**  **R, superior frontal gyrus**  **LC > EC** | | **IR**  **R, postcentral gyrus**  **LC > IC** | | **NW**  **L, precentral gyrus**  **LC > EC** | | **NW**  **R, inferior parietal lobule**  **LC > IC** | | **UR**  **L, postcentral gyrus**  **LC > EC** | | **UR**  **R, inferior parietal lobule**  **LC > IC** | |
| --- | --- | --- | --- | --- | --- | --- | --- | --- | --- | --- | --- | --- | --- | --- | --- | --- |
| **Chronotype**  **Age**  **Education^a^**  **PSQI**  **ESS**  **KSS**  **BDI**  **Alcohol^b^**  **Smoking^c^** | **LC**  -.1  -.01  -.13  -.23  -.22  .32  .46  .41 | **EC**  .16  .11  .14  .30  -.01  .36  -.02  .19 | **LC**  -.40  -.47  -.09  .16  .24  -.25  .27  .37 | **IC**  .11  -.02  .30  .38  -.49  .04  -.29  .13 | **LC**  -.11  .03  -.09  .08  .22  .05  .02  .07 | **EC**  -.19  -.23  -.14  -.16  .26  -.12  -.01  -.31 | **LC**  -.32  -.28  .38  .45  .15  -.33  .21  .21 | **IC**  .21  .25  .16  .15  -.26  -.12  -.11  .42 | **LC**  .14  -.36  .46  .15  .35  .27  -.22  .07 | **EC**  -.19  .06  -.35  -.28  -.43  -.40  .33  .26 | **LC**  -.36  -.43  -.03  -.17  .23  .14  .26  .27 | **IC**  .29  .13  .33  -.12  -.04  -.30  -.38  .21 | **LC**  -.08  -.19  -.10  .33  -.42  -.18  .22  .08 | **EC**  -.35  .40  .01  .03  .43  .22  .03  -.15 | **LC**  -.33  -.45  .02  .22  .35  -.21  .10  .24 | **IC**  .14  -.01  .31  -.04  -.17  -.03  -.15  -.01 |

^a^ expressed in number of school and study years. ^b^ expressed in typical number of standard alcoholic drinks per week. ^c^ expressed in typical number of cigarettes per day, only processed for LCs. R = Right cerebrum. L = Left cerebrum.
