# Supplementary material for: Chronotype Modulates Language Processing-Related Cerebral Activity during Functional MRI (fMRI)
Source: PLoS One. 2015 Sep 23;10(9):e0137197. doi: 10.1371/journal.pone.0137197 (PMC4580315; doi:10.1371/journal.pone.0137197)
Supplement: S3 Table — (DOCX) [file pone.0137197.s003.docx]

**S3 Table**. **Bivariate correlation analysis (Pearson, *r*) of reaction times (RT, mean, ms) of directly (DR), indirectly (IR), unrelated (UR) word pairs and non-words (NW) with anatomical regions of significant BOLD activation (based on Functional Imaging Results, contrast estimates) for early (EC), intermediate (IC) and late (LC) chronotypes. ^a^**

| **Anatomical area of significant effect of chronotype in**  **BOLD activation** (Semantic Priming condition, Directionality) ^b^ | **R, precentral gyrus**  (DR, LC > EC) | | **R, inferior parietal lobule**  (DR, LC > IC) | | **R, superior frontal gyrus**  (IR, LC > EC) | | **R, postcentral gyrus**  (IR, LC > IC) | | **L, precentral gyrus**  (NW, LC > EC) | | **L, precentral gyrus**  (NW, LC > IC) | | **L, postcentral gyrus**  (UR, LC > EC) | | **R, inferior parietal lobule**  (UR, LC > IC) | |
| --- | --- | --- | --- | --- | --- | --- | --- | --- | --- | --- | --- | --- | --- | --- | --- | --- |
| **RT** ^c^ **of Chronotype** (in Semantic Priming condition)  **Pearson, *r*** | **LC** (DR)  -.17 | **EC** (DR)  -.28 | **LC**  **(**DR)  -.33 | **IC**  (DR)  -.19 | **LC**  (IR)  .06 | **EC**  (IR)  .26 | **LC**  (IR)  .05 | **IC**  (IR)  -.80** | **LC**  (NW)  .35 | **EC**  (NW)  .19 | **LC**  (NW)  -.04 | **IC**  (NW)  -.07 | **LC**  (UR)  -.13 | **EC**  (UR)  -.07 | **LC**  (UR)  -.37 | **IC**  (UR)  -.13 |

^a^ The contrast estimates of each significant imaging result in BOLD activation between chronotypes (see Table 3) were correlated with the reaction times of the respective chronotypes during processing of the corresponding semantic priming condition. ^b^ Based on Functional Imaging Results, contrast estimates. Effects of Chronotype. Mixed Effects FLAME, P < .001 (see Table 3). ^c^ Reaction Times (mean, ms).

** Significant correlation coefficient (Pearson, r, P < .01, all other coefficients did not reach significance). R = Right cerebrum. L = Left cerebrum.
